# Development and internal validation of a TLR2-based nomogram for diagnosing pulmonary infection in type 2 diabetes

**DOI:** 10.3389/fendo.2026.1764904

**Published:** 2026-02-18

**Authors:** Yingqian Ma, Fufang Qin

**Affiliations:** Department of General Practice, The Fifth Clinical Medical College of Henan University of Traditional Chinese Medicine (Zhengzhou People’s Hospital), Zhengzhou, China

**Keywords:** diagnostic model, nomograms, pulmonary infection, toll-like receptor 2, type 2 diabetes mellitus

## Abstract

**Objective:**

This study aimed to characterize the expression levels and identify the risk factors associated with Toll-like receptor 2 and 4 (TLR2/4) mRNA in peripheral blood mononuclear cells of patients with type 2 diabetes mellitus (T2DM) complicated with pulmonary infection, and to develop and internally validate a nomogram-based diagnostic model.

**Methods:**

A total of 239 patients with T2DM admitted to our hospital between January and August 2025 were selected. Based on the presence of concurrent pulmonary infection at admission, they were divided into the T2DM group (n=128) and the T2DM with pulmonary infection group (n=111). TLR2/4 mRNA expression levels, general characteristics, and peripheral blood inflammatory markers were compared between the two groups. Predictors were identified using LASSO regression and logistic regression to construct a discriminant model, with receiver operating characteristic (ROC) curves plotted. Internal validation employed 10-fold cross-validation and bootstrap-based optimism correction (B = 1000). Model performance was assessed via Hosmer-Lemeshow tests and decision curve analysis (DCA).

**Results:**

Patients with T2DM and pulmonary infection exhibited significantly elevated levels of fasting blood glucose, inflammatory markers (WBC, NEUT, hsCRP, PCT, ESR), and TLR2/4 mRNA expression, as well as higher rates of invasive procedures, compared with the T2DM group (all P < 0.05). Using LASSO feature selection followed by multivariable logistic regression, a diagnostic nomogram was developed incorporating TLR2, IL-6, TNF-α, ESR, age, and diabetes duration. The nomogram demonstrated excellent discrimination, with an apparent AUC of 0.987. Internal validation confirmed robust performance, yielding a 10-fold cross-validation AUC of 0.980 ± 0.006 and a bootstrap optimism-corrected AUC of 0.980 (B = 1,000).The Hosmer–Lemeshow test indicated good calibration (P > 0.05). DCA showed substantial net clinical benefit across threshold probabilities ranging from 0.10 to 0.60.

**Conclusion:**

TLR2, IL-6, TNF-α, ESR, age, and duration of diabetes can serve as a combined biomarker panel to aid in the early diagnosis of pulmonary infection in T2DM patients at hospital admission. The proposed nomogram demonstrates strong diagnostic performance and potential clinical utility.

## 1 Introduction

Type 2 Diabetes Mellitus (T2DM) is a group of disorders characterized by metabolic dysfunction, with high incidence rates and numerous complications. According to the International Diabetes Federation (2022) report, approximately 536 million people aged 20–79 worldwide had diabetes in 2021, with projections reaching 785 million by 2045 (1). Concurrently, the proliferation of microorganisms, emergence and spread of pathogens, and escalating bacterial resistance have led to a dramatic increase in pulmonary infections (2). Diabetic patients exhibit significantly heightened systemic inflammation and immune deficiencies, making them more susceptible to pulmonary infections (3). Studies indicate that individuals with T2DM face over three times the risk of developing pulmonary infections compared to non-diabetics, with substantially increased rates of complications and mortality following infection (4), imposing a substantial burden on society and public health.

Among innate immune pathways, the Toll-like receptor (TLR) family plays a pivotal role in pathogen recognition and the initiation of inflammatory signaling. Monocytes and macrophages, as key effector cells of the innate immune system, rely on TLR-mediated recognition of bacterial components to trigger downstream immune activation (5). Toll-like receptor 2 (TLR2) and Toll-like receptor 4 (TLR4) are particularly important in sensing gram-positive and gram-negative bacterial pathogens, respectively, and their activation shapes the magnitude and direction of inflammatory responses (6). Dysregulated expression or overactivation of TLR2/4 has been implicated in exaggerated inflammatory states and infection-related complications among individuals with metabolic disorders, including T2DM (7).

Systematic studies on the expression profiles of TLR2 and TLR4 in peripheral blood mononuclear cells (PBMCs) from patients with type 2 diabetes mellitus (T2DM) complicated by pulmonary infection remain scarce. Furthermore, there is a lack of integrated diagnostic models combining TLR-related immune characteristics with conventional clinical parameters to enhance the early detection rate of pulmonary infection in this population. To date, no standardized diagnostic tools suitable for clinical application have been established, nor have validated thresholds based on Toll-like receptors expression been developed.

Therefore, this study focuses on patients with T2DM complicated by pulmonary infection and examines their clinical characteristics, inflammatory biomarkers, and TLR2/4 mRNA expression levels in PBMCs. We aimed to evaluate the diagnostic value of inflammation-related biomarkers, including TLR2 and TLR4, in type 2 diabetes patients with pulmonary infection at admission, and to develop and internally validate a parsimonious diagnostic nomogram.

## 2 Methods

### 2.1 Study design

This retrospective cohort study enrolled patients with T2DM admitted to our hospital between January 2025 and August 2025. Participants were categorized into the T2DM group and the T2DM with pulmonary infection group (T2DM-PI group) based on the presence of concurrent pulmonary infection at admission. All infections were community-acquired and present at admission. The study protocol was approved by the Medical Ethics Committee of Zhengzhou People’s Hospital (Ethics Approval No.: YX-2024-073-01). All research procedures adhered to the Declaration of Helsinki and relevant ethical guidelines.

### 2.2 Inclusion and exclusion criteria

#### 2.2.1 Inclusion criteria

1 Patients with type 2 diabetes meeting the criteria of the Chinese Diabetes Prevention and Treatment Guidelines (2024 Edition) (8);
2 Diagnostic criteria for community-acquired pneumonia (CAP): Patients were diagnosed with CAP based on new-onset cough and sputum production or worsening of existing respiratory symptoms, fever, and physical signs of pulmonary consolidation. Supportive laboratory findings included a peripheral WBC count >10×10^9^/L or <4×10^9^/L. Chest imaging was required to demonstrate new infiltrates, consolidation, or ground-glass changes, with or without pleural effusion. Microbiological evidence was obtained through sputum smear or culture when available. All pneumonia cases in this study were confirmed at admission and classified as CAP.
3 Patients who have not received anti-infective therapy within 2 weeks prior to study enrollment.

#### 2.2.1 Exclusion criteria

1 Patients with acute diabetic complications such as diabetic ketoacidosis or hyperosmolar coma;
2 Patients with infections in other body areas, including the abdomen, urinary tract, or skin;
3 Patients with hospital-acquired pneumonia (HAP) or ventilator-associated pneumonia (VAP);
4 Patients with liver or kidney dysfunction, malignant tumors, tuberculosis, myocardial infarction, hematological disorders, or conditions affecting platelet or lymphocyte levels;
5 Patients with septic shock, coma, impaired consciousness, or those in the terminal stage of clinical disease;
6 Patients with psychiatric disorders, inability to communicate, or inability to cooperate.

### 2.3 Data collection

Demographic and clinical data were retrospectively extracted from the electronic medical record system. This included patient gender, age, and body mass index (BMI); duration of type 2 diabetes; smoking and alcohol consumption history; length of hospital stay; and occurrence of invasive procedures. Documented comorbidities comprised hypertension, hyperlipidemia, coronary heart disease, and cerebral infarction.

On the first day of admission, fasting venous blood samples collected upon waking are sent to the laboratory for testing. Tests included complete blood count, fasting blood glucose (FBG), albumin (ALB), glomerular filtration rate (GFR), total cholesterol (CHOL), triglycerides (TG), interleukins (IL-1β, IL-6), tumor necrosis factor-alpha (TNF-α), high-sensitivity C-reactive protein (hsCRP), erythrocyte sedimentation rate (ESR), and procalcitonin (PCT). Additionally, the neutrophil-to-lymphocyte ratio (NLR) and Systemic Immune-Inflammation Index (SII) were calculated.

We employed real-time quantitative polymerase chain reaction (RT-qPCR) to detect TLR2/4 mRNA expression. On the morning of the first day of admission, 2 mL of fasting venous blood was collected from each enrolled patient and sent to our hospital’s laboratory for analysis. Quantitative measurements of TLR2 and TLR4 mRNA expression were performed using the Thermo Fisher ABI 7500 real-time PCR system. Primer sequences were designed and synthesized by BGI, see **Supplementary Table S1** for details. GAPDH was used as the internal reference, and the relative expression levels of TLR2 and TLR4 mRNA were calculated using the 2^(-ΔΔCt) method.

### 2.4 Statistical analysis

All statistical analyses were performed using SPSS software (version 23.0) and R software (version 4.3.2). All statistical tests were two-tailed, and a P value < 0.05 was considered statistically significant. Categorical variables are expressed as numbers and percentages [n (%)], and comparisons between groups were performed using the chi-square test. Continuous variables were assessed for normality prior to analysis. Normally distributed variables were expressed as mean ± standard deviation (SD) and compared using the independent-samples t-test. Non-normally distributed variables were summarized as median with interquartile range [median (P25, P75)] and compared using the Wilcoxon rank-sum test (Mann–Whitney U test).

Candidate predictors were first selected using least absolute shrinkage and selection operator (LASSO) regression to reduce redundancy and mitigate overfitting. Variables retained by LASSO were then entered into a multivariable logistic regression model to identify independent diagnostic factors associated with pulmonary infection. A nomogram was subsequently developed based on the multivariable model coefficients to enable individualized risk estimation and diagnostic support. Model discrimination was assessed using the area under the receiver operating characteristic (ROC) curve (AUC). To evaluate model robustness and reduce potential overfitting, internal validation was performed by calculating the 10-fold cross-validation AUC, and further estimating the bootstrap optimism-corrected AUC using 1,000 bootstrap resamples (B = 1,000). Model calibration was evaluated by visual inspection of calibration plots and statistically examined using the Hosmer–Lemeshow goodness-of-fit test. The clinical utility of the nomogram was assessed using decision curve analysis (DCA), which quantifies the net benefit across a range of threshold probabilities and compares the model with the default strategies of treating all patients and treating none.

## 3 Results

### 3.1 Baseline characteristics

Patients with T2DM and pulmonary infection had significantly higher age, body temperature, and diabetes duration compared with those with T2DM alone (p < 0.05). In addition, the proportion of patients undergoing invasive procedures was markedly higher in the pulmonary infection group than in the non-infection group (36.94% vs. 11.72%, p < 0.001, **Table 1**).

**Table 1:** Baseline characteristics.

Variable	T2DM group (n=128)	T2DM-PI group (n=111)	Z/*χ^2^/t* value	P value
Age (year)	65.41 ± 11.49	69.77 ± 12.56	-2.801	0.006
Sex, n (%)			2.972	0.085
Male	81(63.28)	58(52.25)		
Female	47(36.72)	53(47.75)		
Smoking, n (%) Alcohol consumption, n (%) Comorbidities, n (%)	31(24.22)	26(23.42)	0.021	0.886
Alcohol consumption, n (%)	31(24.22)	20(18.02)	1.362	0.243
Comorbidities, n (%)				
Hypertension	63(49.22)	61(54.95)	0.784	0.376
Hyperlipidemia	60(46.88)	41(36.94)	2.406	0.121
Coronary heart disease	66(51.56)	51(45.95)	0.750	0.386
Cerebral infarction	27(21.09)	35(31.53)	3.371	0.066
BMI (kg/m^2^)	25.33 ± 3.42	24.92 ± 4.08	0.859	0.391
Length of hospital stay (days)	7(5.,9)	7 (5,10)	-1.592	0.111
Duration of diabetes (years)	10(5,17)	13(10,20)	42.817	0.027
Body temperature (°C)	36.3(36.2,36.5)	37.7(37.2,38.2)	-11.08	<0.001
Invasive procedures, n (%)	15(11.72)	41(36.94)	21.073	<0.001

BMI, body mass index.

### 3.2 Peripheral blood parameter levels

Patients with T2DM complicated by pulmonary infection exhibited significantly higher levels of WBC, NEUT, LYMPH, NLR, SII, FBG, IL-6, TNF-α, hsCRP, PCT, and ESR compared to patients with T2DM alone (p < 0.05, **Table 2**).

**Table 2:** Peripheral blood parameter levels.

Variable	T2DM group (n=128)	T2DM-PI group(n=111)	Z/t value	P value
WBC(×10^9^/L)	6.19(5.29,7.30)	8.34(6.26,10.08)	-5.497	<0.001
NEUT (×10^9^/L)	3.84(3.28,4.81)	5.09(3.73,7.73)	-5.173	<0.001
LYMPH (×10^9^/L)	1.59(1.29,2.02)	1.32(0.97,1.87)	-3.151	0.002
PLT(×10^9^/L)	129.39(99.04,167.09)	152.00(112.64,207.58)	-0.363	0.717
NLR	2.23(1.78,3.20)	3.72(2.32,6.46)	-5.536	<0.001
SII	478.13(330.07,699.48)	794.03(449.83,1375.66)	-5.110	<0.001
FBG (mmol/L)	7.78 (6.57, 9.39)	8.05(7.13,9.60)	-2.038	0.042
ALB(g/L)	43.74 ± 3.64	40.84 ± 3.93	5.912	<0.001
GFR (ml/min)	130.69 (110.65, 153.06)	122.97(99.21,152.28)	-0.681	0.496
CHOL(mmol/L)	3.95(3.33,5.29)	4.00(3.49,4.71)	-0.426	0.670
TG (mmol/L)	1.27 (0.86, 2.18)	1.17(0.96,1.65)	-1.206	0.228
IL-1β (pmol/L)	6.97(6.24,7.71)	6.97(6.24,8.07)	-0.860	0.390
IL-6 (pmol/L)	9.54 (7.71,11.74)	17.50(13.03,22.75)	-9.278	<0.001
TNF-α (pmol/L)	6.73(5.87,7.34)	9.18(7.71,9.91)	-8.948	<0.001
hsCRP(mg/L)	1.22(0.62,2.99)	10.77(3.47,34.04)	-9.232	<0.001
ESR (mm/h)	6.15 (4.93, 9.35)	27.50(17.50,37.10)	-10.240	<0.001
PCT (ng/L)	29.00(21.00,41.00)	60.00(37.00,96.00)	-7.179	<0.001

WBC, white blood cell count; NEUT, Neutrophils; LYMPH, Lymphocytes; PLT, platelet counts; NLR, NEUT/LYMPH SII:(PLT*NEUT)/LYMPH; FBG, fasting blood glucose; ALB, albumin; GFR, glomerular filtration rate; CHOL, total cholesterol; TG, triglycerides; interleukins (IL-1β; IL-6); TNF-α, tumor necrosis factor-alpha; hsCRP, high-sensitivity C-reactive protein; ESR, erythrocyte sedimentation rate; PCT, procalcitonin.

### 3.3 Expression levels of TLR2 and TLR4 mRNA in PBMCs

The expression levels of TLR2 and TLR4 mRNA in peripheral blood mononuclear cells were significantly higher in patients with T2DM and pulmonary infection than in those without infection (p < 0.05, **Table 3**).

**Table 3:** Expression levels of TLR2 and TLR4 mRNA in PBMCs.

Variable	T2DM group (n=128)	T2DM-PI group (n=111)	*Z* value	P value
TLR2mRNA	2.845(1.443,7.203)	6.884(2.795,16.625)	-4.545	<0.001
TLR4 mRNA	1.117(0.537,3.247)	1.824(0.931,6.405)	-3.788	<0.001

TLR2, Toll-like receptors 2, TLR4, Toll-like receptors 4.

### 3.4 Feature selection of diagnostic predictors for pulmonary infection in T2DM patients using LASSO regression

**Figure 1:**
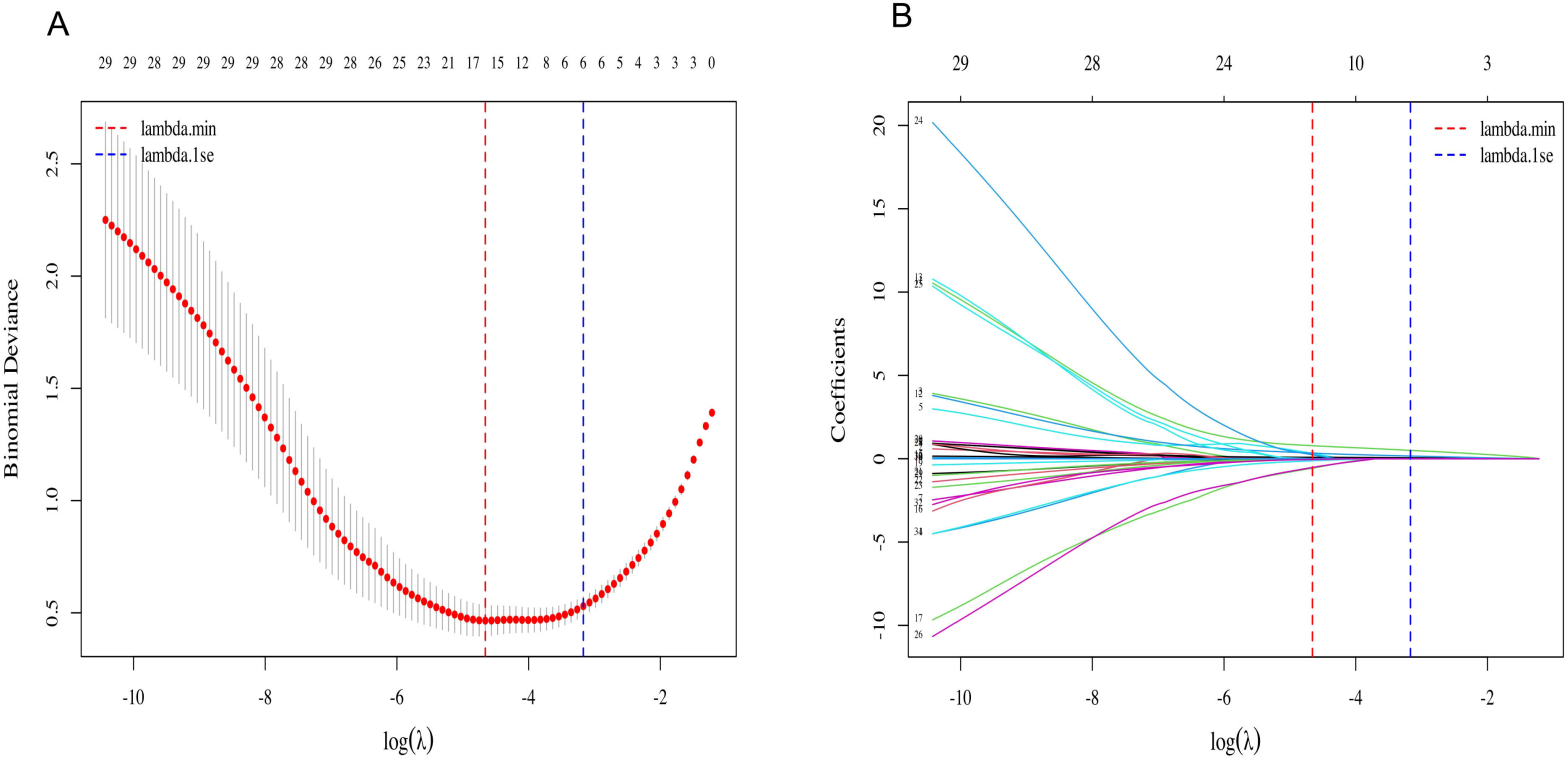
LASSO regression for screening diagnostic variables of pulmonary infection in type 2 diabetes patients. **(A)** Ten-fold cross-validation curve for selecting the optimal penalization parameter (λ) based on binomial deviance. **(B)** LASSO coefficient profiles of candidate predictors as a function of log(λ). Each curve represents a predictor.

T2DM patients with concomitant pulmonary infection at admission were defined as the dependent variable. Candidate clinical characteristics and inflammation-related biomarkers were included as independent variables. LASSO regression was applied for feature selection, and the optimal penalization parameter (λ) was determined using 10-fold cross-validation with binomial deviance as the evaluation criterion. Cross-validation results indicate that binomial deviance exhibits a typical U-shaped trend with respect to log(λ), reaching its minimum at lambda.min = 0.0095. To obtain a more parsimonious and robust model, we selected λ = 0.0420 (lambda.1se) according to the 1-standard-error rule (**Figure 1A**). At the selected lambda.1se, six predictors with non-zero coefficients were retained for subsequent multivariable logistic regression, including TLR2, TLR4, ESR, PCT, IL-6 and TNF-α (**Figure 1B**).

### 3.5 Independent risk factors for pulmonary infections in T2DM

Using the six candidate predictors selected by LASSO regression, multivariable logistic regression identified TLR2, IL-6, TNF-α, and ESR as independent diagnostic factors associated with pulmonary infection at admission in patients with type 2 diabetes. Higher levels of TLR2 (OR = 1.102, 95% CI: 1.032–1.177, P = 0.004), IL-6 (OR = 3.072, 95% CI: 1.658–5.692, P < 0.001), TNF-α (OR = 1.923, 95% CI: 1.394–2.651, P < 0.001), and ESR (OR = 1.170, 95% CI: 1.093–1.252, P < 0.001) were significantly associated with increased odds of pulmonary infection. To enhance clinical applicability, age and duration of diabetes were mandatorily retained in the final model; age was not statistically significant after adjustment (OR = 1.063, 95% CI: 0.974–1.161, P = 0.168), whereas duration of diabetes showed an inverse association (OR = 0.848, 95% CI: 0.722–0.996, P = 0.045). The final diagnostic model therefore included TLR2, IL-6, TNF-α, ESR, Age, and Duration of diabetes (**Table 4**).

**Table 4:** Multivariate logistic regression analysis of pulmonary infection in patients with Type 2 Diabetes Mellitus.

Variable	B value	Wald value	P value	OR	95%CI
TLR2	0.097	8.347	0.004	1.102	1.032~1.177
IL-6	1.122	12.717	<0.001	3.072	1.658~5.692
TNF-α	0.654	15.906	<0.001	1.923	1.394~2.651
ESR	0.157	20.556	<0.001	1.170	1.093~1.252
Age	0.062	1.901	0.168	1.063	0.974~1.161
Duration of diabetes	-0.165	4.034	0.045	0.848	0.722~0.996

### 3.6 Nomogram development and internal validation for identifying pulmonary infection at admission

**Figure 2:**
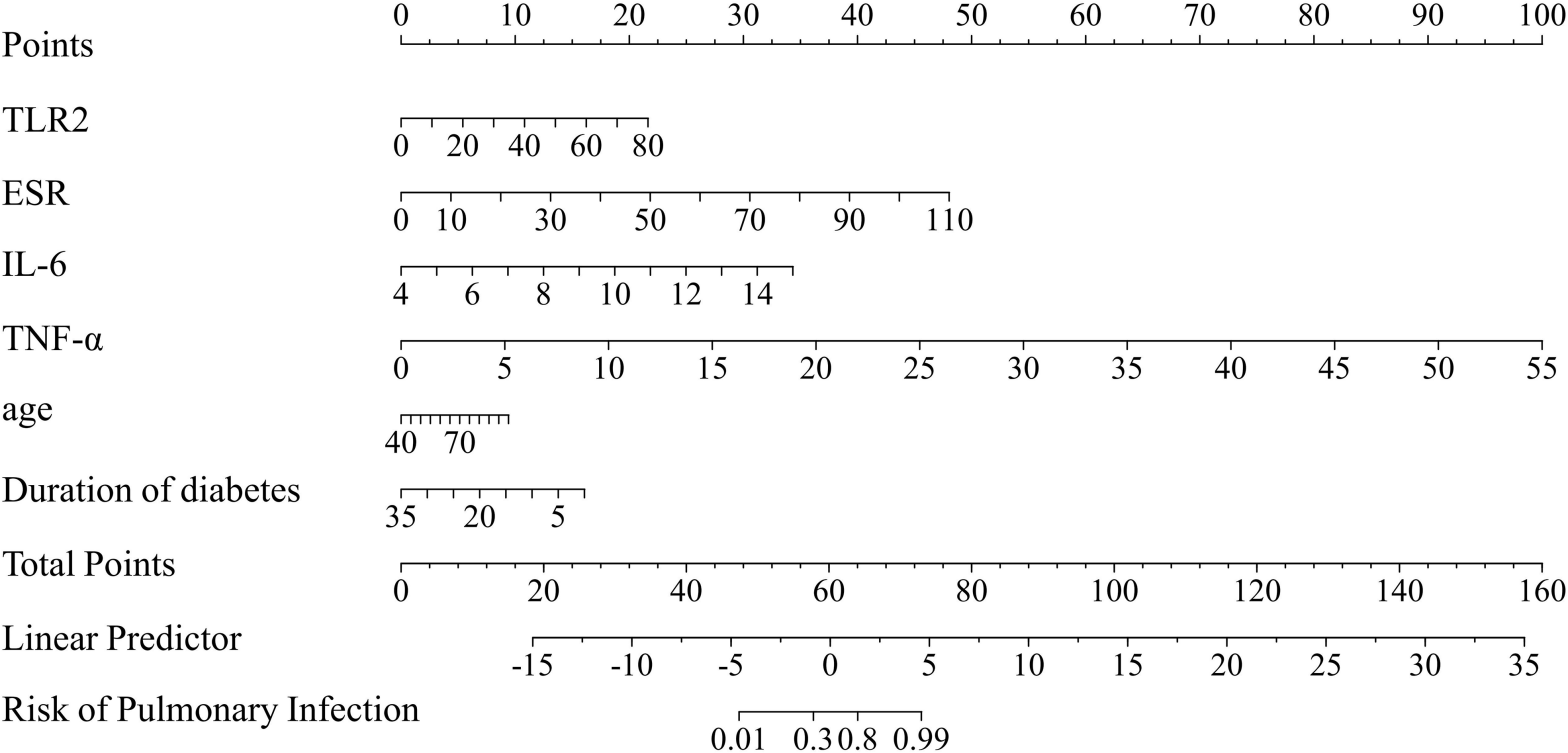
Diagnostic nomogram for identifying pulmonary infection present at admission in patients with type 2 diabetes mellitus. Each factor is assigned a point value; the total score corresponds to the probability of a confirmed diagnosis of pulmonary infection.

Based on the multivariable logistic regression results, six independent indicators (TLR2, IL-6, TNF-α, ESR, Age, and Duration of diabetes) were incorporated to develop a diagnostic nomogram for diagnosing pulmonary infection present at admission in patients with T2DM (**Figure 2**). The nomogram translates regression coefficients into a point-based scoring system: each predictor value is mapped to a corresponding score on the “Points” axis, and the sum of individual scores yields the “Total Points”, which is then converted into the predicted risk of pulmonary infection. Overall, the inflammatory biomarkers (TLR2, ESR, IL-6, and TNF-α) accounted for a substantial proportion of the total score range, whereas diabetes duration showed an inverse contribution consistent with its negative association in the model. A higher total score indicates a higher likelihood of pulmonary infection.

### 3.7 Nomogram performance, calibration, and clinical utility

**Figure 3:**
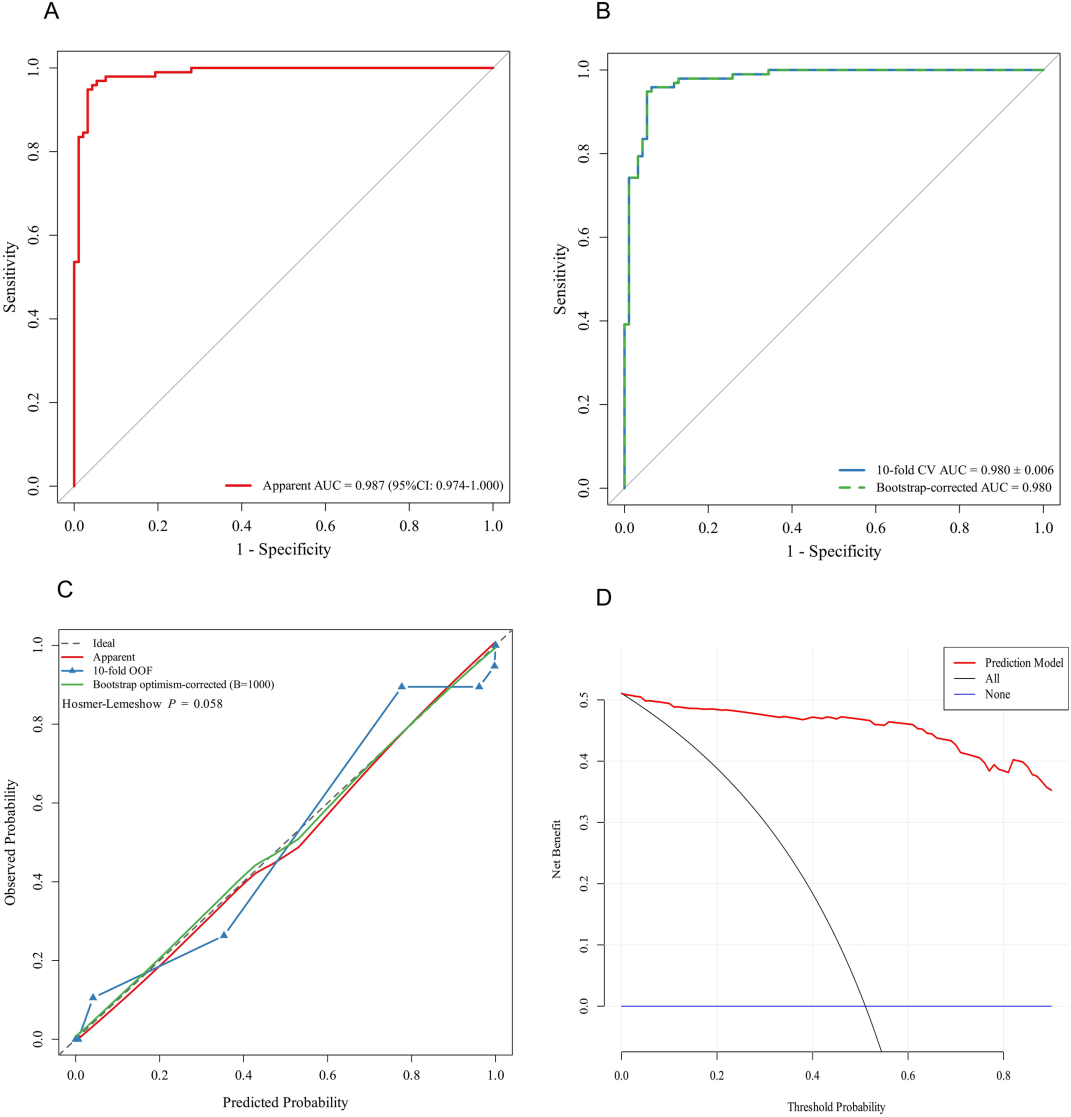
Development and internal validation of a diagnostic model for pulmonary infection in patients with type 2 diabetes mellitus. **(A)** ROC curve. **(B)** Internal validation ROC curves, including 10-fold cross-validation (out-of-fold) performance and bootstrap optimism-corrected performance. **(C)** Calibration curve of Hosmer-Lemeshow test. **(D)** Decision Curve Analysis (DCA).

A nomogram was established based on the final multivariable logistic regression model incorporating TLR2, IL-6, TNF-α, ESR, age, and duration of diabetes. The ROC curve demonstrated excellent discrimination, with an apparent AUC of 0.987 (95% CI: 0.974–1.000) (**Figure 3A**). The model remained robust on internal validation, yielding a 10-fold cross-validation AUC of 0.980 ± 0.006, and a bootstrap-corrected AUC of 0.980 (**Figure 3B**). Calibration plots suggested an overall agreement between predicted and observed probabilities, with comparable calibration patterns across the apparent fit, 10-fold out-of-fold estimates, and optimism-corrected bootstrap calibration (B = 1,000). The Hosmer-Lemeshow test results indicate that the predicted values align with the observed values (P = 0.058) (**Figure 3C**). For clinical utility, decision curve analysis (DCA) was interpreted within a clinically plausible threshold probability range (0.10–0.60). Across this interval, the nomogram provided a higher net benefit than the default strategies of treating all patients or treating none, supporting its potential usefulness for admission-based diagnostic decision-making and risk stratification (**Figure 3D**).

## 4 Discussion

T2DM is a highly heterogeneous and complex metabolic disorder characterized by dysregulated signaling pathways, impaired immune homeostasis, and dysfunction of multiple immune cell populations (9). Aberrant cytokine responses further contribute to immune vulnerability, rendering individuals with T2DM more prone to pathogen invasion and subsequent infections (10). Risk stratification tools, such as visualized prediction charts, offer simplified and intuitive models that facilitate rapid clinical decision-making at the point of care (11). This retrospective study used multivariable logistic regression, together with the mandatory inclusion of clinically relevant variables, to identify six predictors—TLR2, IL-6, TNF-α, ESR, age, and diabetes duration—and to develop a nomogram for admission-based identification of pulmonary infection in patients with type 2 diabetes mellitus. The model showed excellent discrimination, with an apparent AUC of 0.987. Internal validation further supported its robustness, yielding a 10-fold cross-validation AUC of 0.980 ± 0.006 and a bootstrap optimism-corrected AUC of 0.980.

Our findings demonstrated that patients with T2DM who developed pulmonary infections exhibited significantly higher fasting blood glucose levels compared with those with T2DM alone (P < 0.05). Persistent hyperglycemia creates a metabolic environment that not only favors the growth and replication of pathogenic microorganisms but also impairs innate immune responses by diminishing the secretion of interferons and key proinflammatory cytokines. Such dysregulation has been shown to accelerate macrophage senescence, thereby further compromising host defense mechanisms (12, 13). In 2014, the Systemic Inflammation Index (SII) was first introduced as an integrated biomarker capable of capturing both local immune responses and overall systemic inflammatory burden in the human body (14). Elevated SII levels generally indicate intensified inflammatory activity and have been increasingly utilized as an auxiliary indicator for evaluating disease severity. Nevertheless, dependence on SII alone may result in an incomplete or even misleading assessment of the patient’s inflammatory status. A more accurate appraisal requires synthesizing SII with clinical manifestations, radiological findings, erythrocyte sedimentation rate (ESR), and specific cytokine profiles such as interleukins (15).

The results of this study showed that the expression levels of IL-6, TNF-α, and ESR were significantly higher in patients with T2DM complicated by pulmonary infection than in those with T2DM alone (P < 0.05). IL-6 is a key proinflammatory cytokine involved in the host response to infection. Following pathogen invasion, bacterial components such as lipopolysaccharides can activate immune cells through receptors including TLR4, thereby inducing the release of IL-6 (16). In pulmonary infections, alveolar macrophages and infiltrating neutrophils constitute the primary sources of IL-6. Macrophages can rapidly recognize pathogens and secrete IL-6 directly, while neutrophils modulate IL-6 production indirectly through the release of regulatory mediators such as IL-10 (17). Moreover, a hyperglycemic state may further amplify inflammatory signaling pathways and disrupt the pulmonary microenvironment, ultimately promoting excessive IL-6 expression (18). TNF-α is a pivotal multifunctional inflammatory mediator produced by a variety of immune cells. During pathogen invasion, TLR-mediated signaling pathways trigger downstream inflammatory cascades, leading to the activation of NF-κB and subsequently promoting TNF-α gene transcription (19). In addition, alveolar macrophages exposed to inflammatory stimuli can secrete granulocyte colony-stimulating factor, which markedly enhances TNF-α expression and intensifies the local inflammatory response (20). ESR is a fundamental laboratory indicator that reflects the extent of tissue injury and systemic inflammatory activity and is widely used as an effective marker in clinical assessment and management. Elevated ESR is commonly observed in patients with pulmonary infections. In a study by Qaisieh et al. (21), more than 80% of patients with COVID-19 exhibited increased ESR levels, which were further associated with lymphopenia and greater severity of pulmonary involvement.

TLR2 and TLR4 are central members of the Toll-like receptor family and play essential roles in recognizing pathogen-associated molecular patterns (PAMPs) and initiating innate immune responses. Upon activation, these receptors trigger downstream signaling pathways that induce the production of proinflammatory cytokines and type I interferons, thereby contributing to the early defense against invading pathogens (5). TLR2 primarily recognizes components of Gram-positive bacteria, such as bacterial lipoproteins and lipoteichoic acid, as well as fungal structures including zymosan. Functionally, it forms heterodimers with TLR1 or TLR6 to mediate downstream signaling (22). In contrast, TLR4 predominantly detects lipopolysaccharides from Gram-negative bacteria but can also respond to certain viral proteins and damage-associated molecular patterns released from host tissues (23). Moreover, both TLR2 and TLR4 induce early inflammatory responses through MyD88-dependent pathways (5). This study found that the expression levels of TLR2 and TLR4 mRNA in peripheral blood mononuclear cells from the infected group were significantly higher than those in the non-infected group (P < 0.05), suggesting that TLR2 and TLR4 may be involved in the pathological process of pulmonary infection in patients with T2DM. This finding is consistent with previous studies (24), suggesting that aberrant activation of TLR2 and TLR4 may intensify inflammatory responses and contribute to immune dysregulation. Excessive activation of these receptors can lead to the overproduction of proinflammatory cytokines, potentially triggering a “cytokine storm” that exacerbates pulmonary tissue injury (25). Therefore, TLR2 and TLR4 not only serve as key molecules in pathogen recognition but also function as critical regulators of inflammatory processes. Although TLR4 has a well-established biological role in innate immune activation, it failed to maintain statistical significance after multivariate adjustment in this cohort and was therefore excluded from the final model. This may be attributed to TLR4’s insufficient independent contribution in this study, with its effects potentially partially “absorbed” by inflammatory markers such as TLR2, IL-6, and TNF-α. Concurrently, correlations among markers within the inflammatory pathway and limited statistical power due to the single-center sample size cannot be ruled out. Future validation of TLR4’s independent role in multi-center, independent cohorts remains necessary.

During model development, we strived to balance statistical parsimony, interpretability, and real-world applicability. Although age did not demonstrate statistical significance after adjustment in the multivariate logistic regression model (P = 0.168), it remains a clinically important indicator associated with infection susceptibility and immune aging, and is readily obtainable in routine clinical practice. Therefore, to enhance clinical applicability, the final diagnostic model mandatorily retained both age and diabetes duration as indicators to improve the model’s transferability across different populations and healthcare settings. Notably, diabetes duration exhibited a negative correlation with pulmonary infection at admission (OR = 0.848, P = 0.045). This finding should not be interpreted as indicating a protective effect of prolonged disease duration; rather, it likely reflects differences in disease spectrum and healthcare-seeking behavior among hospitalized patients. Patients with longer disease duration typically receive more regular follow-up and comprehensive management, potentially seeking medical care earlier when respiratory symptoms arise. This may reduce the likelihood of developing a confirmed pulmonary infection requiring hospitalization. Conversely, patients with shorter recorded disease duration may include newly diagnosed but previously uncontrolled cases, who are more likely to present with severe infection at the time of initial consultation.

This study focuses on the early identification and diagnostic support of pulmonary infection at admission in patients with type 2 diabetes mellitus (T2DM), enabling timely initiation of targeted evaluation and management during the early stage of hospitalization. The nomogram developed from multivariable logistic regression integrates inflammation- and immunity-related biomarkers (TLR2, IL-6, TNF-α, and ESR) together with clinically meaningful and readily available variables (age and duration of diabetes), thereby providing an individualized quantitative estimate of the probability of pulmonary infection at admission. This tool may assist clinicians in admission-based risk stratification and diagnostic decision-making, and facilitate earlier targeted interventions and comprehensive management when clinically indicated, potentially reducing missed diagnoses and improving diagnostic efficiency. The model demonstrated high discrimination in both the apparent analysis and internal validation, and showed stable calibration patterns and potential clinical benefit in calibration and decision curve analyses. Notably, DCA indicated favorable net benefit within a clinically actionable threshold probability range of 0.10–0.60, supporting its potential utility in real-world admission settings.

However, this study still has several limitations. First, the model was constructed based on a single-center cohort with a limited sample size (n=239). Despite employing Lasso feature selection and internal validation, its performance may be overestimated due to insufficient heterogeneity and potential spectrum effects, posing a risk of overfitting. Second, as the outcome reflects acute inflammatory responses at admission, model performance may vary across populations with different disease severity, clinical workflows, or laboratory platforms. Third, this study has only undergone internal validation and lacks independent external cohort validation. The model’s generalization capability requires further confirmation across multiple centers, different time windows, and diverse populations to enhance cross-scenario stability. Collectively, future studies should perform external validation and prospective real-world evaluations in larger multicenter cohorts, incorporate subgroup analyses and dynamic biomarkers, and further refine the model while delineating its clinical scope.

## 5 Conclusions

In summary, pulmonary infection is a common complication in patients with type 2 diabetes mellitus, making its timely identification upon hospital admission critically important. This study identified TLR2, IL-6, TNF-α, and ESR as key inflammatory diagnostic markers and developed a practical nomogram incorporating age and duration of diabetes to support diagnosis at admission. The model demonstrated excellent discriminatory power and stable internal validation performance. Decision curve analysis indicated its potential clinical benefit within a clinically actionable threshold probability range. However, external validation through an independent multicenter cohort is still required to further confirm the model’s adaptability and clinical utility across different healthcare settings.

## Data Availability

The raw data supporting the conclusions of this article will be made available by the authors, without undue reservation.
