# Supplementary material for: Development and internal validation of a TLR2-based nomogram for diagnosing pulmonary infection in type 2 diabetes
Source: Front Endocrinol (Lausanne). 2026 Feb 18;17:1764904. doi: 10.3389/fendo.2026.1764904 (PMC12956657; doi:10.3389/fendo.2026.1764904)
Supplement: Supplementary file 1 [file Table1.docx]

| **Table S1 Primer Sequences** | | |
| --- | --- | --- |
| Primer | Primer Sequence(5'→3') | Length(bp) |
| TLR2-F | GGCGTTCTCTCAGGTGACTG | 122 |
| TLR2-R | CCCTGTCTTCCTGCCTTCAC |  |
| TLR4-F | GCGTGGAGGTGGTTCCTAAT | 127 |
| TLR4-R | CTGCCTAAATGCCTCAGGGG |  |
| GAPDH-F | TCGGAGTCAACGGATTTGGT | 181 |
| GAPDH-R | TTCCCGTTCTCAGCCTTGAC |  |
